# Immune Supplements Under the Magnifying Glass: An Expert Panel Develops Priorities and Evidence-Based Recommendations for Future Research Regarding Dietary Supplements

**DOI:** 10.1089/jicm.2022.0800

**Published:** 2023-04-11

**Authors:** Cindy Crawford, LaVerne L. Brown, Rebecca B. Costello, Patricia A. Deuster

**Affiliations:** ^1^ Consortium for Health and Military Performance, Department of Military and Emergency Medicine, F. Edward Hébert School of Medicine, Uniformed Services University, Bethesda, MD, USA.; ^2^ Henry M. Jackson Foundation for the Advancement of Military Medicine, Bethesda, MD, USA.; ^3^ Office of Dietary Supplements, National Institutes of Health, Bethesda, MD, USA.

## Introduction

Dietary supplements advertised to support or boost the immune system are intended to target healthy consumers wishing to maintain or optimize their health, or resist, recover, or grow from a challenge or life stressor they may be facing. The ultimate goal could be not getting sick with, or recovering more quickly from, such ailments as the common cold, cough, congestion, fever, body aches, and influenza. Dietary supplements are not meant to prevent or treat disease but to supplement the diet.^1^ Yet, for reasons beyond the scope of this letter, much of the research on dietary supplements tends to explore treatment outcomes rather than outcomes relevant to maintaining and enhancing health, within a resilience framework.^2^

The authors performed a scoping review of the market to identify the frequently listed ingredients contained in immune boosting dietary supplements,^3^ analyzed select products,^4^ performed a systematic review to evaluate the evidence surrounding such claims made,^3^ and convened a research expert panel to develop priorities and evidence-based recommendations for future research. The end product would be information so the public can make evidence-informed decisions when choosing products.

## Methods

Eleven diverse panelists with no conflicts or financial interests to disclose participated in a research expert panel jointly sponsored by the Office of Dietary Supplements, National Institutes of Health, and the Consortium for Health and Military Performance, Uniformed Services University. A white paper detailing the systematic review evidence for select dietary supplement ingredients in preserving and protecting the immune system in otherwise healthy individuals who may be exposed to stressors (e.g., winter season, extreme exercise, academic stress, and air travel) was shared with panelists. Panelists independently reviewed the evidence, rated the importance of specific elements for research on dietary supplements in general, answered specific research questions posed through systematic review, and prioritized future research directions for specific dietary supplement ingredients.

The authors collected the initial ratings and produced a deidentified summary report to represent the spread of agreement. Panelists convened virtually to review the distribution of ratings, discuss areas of diverging opinion, and finally rerate anonymously.

## Results

Most panelists agreed on the importance of disclosing specific details when planning, conducting, and reporting on dietary supplement research; they rated details as “absolutely important,” with scores ranging from 7 to 9 on a 9-point scale. Some level of uncertainty was raised surrounding the importance of 6 of the 21 details asked about. Through discussion, panelists agreed to the importance of these items to ensure rigor, reproducibility, and ultimately, generalizability to the end-user (Table 1).

**Table 1.** Importance of Specific Items in General When Planning, Conducting, and Reporting on Dietary Supplement Research in the Focus Area of the Research Questions Posed Through Systematic Review Conducted

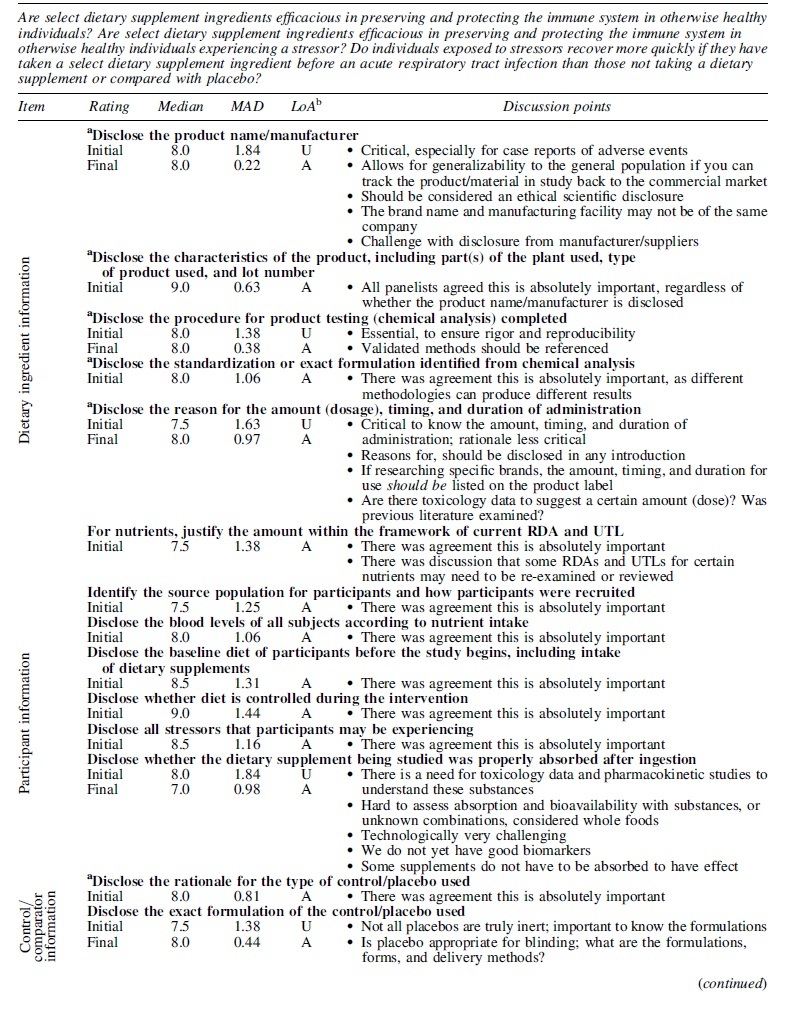 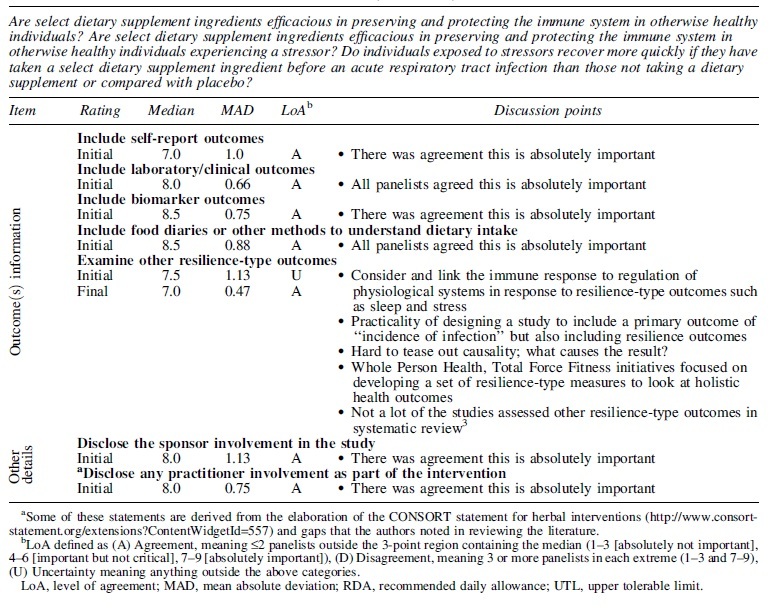

Panelists' opinions of how they would answer the research questions posed through systematic review varied across ingredients evaluated. Relying on what they believed could be reasonably claimed based on the evidence and what additional evidence or studies would be needed to make additional claims, panelists developed priorities for the ingredients evaluated, discussed barriers, and rated whether it would be appropriate to invest resources in clinical trials at this time (Table 2). Zinc received the highest priority rating for future research.

**Table 2.** Priorities for Future Research Within the Context of Systematic Review Research Questions, Evaluation, and Findings

Priorities for future research^a^			What can be said about the evidence^3^	What's needed to say more; barriers to research and resource needs	Approp. to invest in clinical trials now^b^
Statistical method	Initial rating	Final rating			
**Echinacea (*E. angustifolia* and *E. purpurea*)**					
Median	6.5	7.0	Individual studies show few statistically significant results, but overall, results are consistent in showing positive trends of less risk of getting an acute respiratory tract infection among otherwise healthy individuals who may be experiencing certain life stressors, taking various Echinacea formulations prophylactically	*What's needed to say more* • More RCT research on stressed individuals to confirm effect (e.g., winter season stress and/or relevant viral challenge) • Standardized formulation for relevance to consumer options (tablet vs. liquid extract vs. tea); compare lower dose ranges (e.g., drinking Echinacea tea) vs. higher doses (e.g., ingesting an Echinacea extract) • Compared with placebo • Outcomes including number of infections per group, severity, duration of sick days • Duration over months *Barriers to research and resource needs* • Various species and types of extracts available on the commercial market • Botanical composition not fully characterized or quantified across cultivars; no pharmacokinetic data to understand the various forms • Incomplete toxicology or mechanisms of action for safety • No tolerable upper limit • Funding and size of the study required for which formulation of Echinacea and involving which types of stressors individuals may be experiencing vs. a relevant viral challenge	6.0
MAD	1.50	0.89			0.67
LoA^c^	U	U			U
**Elderberry *(Sambucus nigra* L.)**					
Median	6.0	—	Only one study has been published on healthy adults taking elderberry dietary supplements prophylactically during air travel, showing reduction in cold duration and severity	*Barriers to research and resource needs* • The composition of elderberry is unknown • Reported analytical chemistry methods are not typically validated among matrices and with an appropriate reference standard • Understanding of drug/elderberry interactions • Classic toxicology data not available • Lack of dose ranging studies • No tolerable upper limit as a “functional food”	6.0
MAD	1.06	—			1.35
LoA	A	—			U
**Garlic (*Allium sativum*)**					
Median	5.5	5.0	Few studies available show that adults taking garlic supplements throughout cold and flu season may experience less episodes or symptoms overall	*What's needed to say more* • More RCT research involving larger study samples • Involving native garlic, including composition of product • Compared with placebo • Outcomes including number of infections per group, severity, duration of sick days; biomarkers that are clinically relevant • Duration over months *Barriers to research and resource needs* • Need better composition data on native garlic • Variability in different garlic products • Biomarkers that are clinically relevant • Understanding of potential adverse events and allergic reactions • Funding and size of the studies	5.0
MAD	1.38	0.69			0.73
LoA	U	A			A
			
**Vitamin A**					
Median	4.5	4.0	Studies conducted in countries with high prevalence of vitamin A deficiency, in children younger than 10 years. Other reviews point to different effects dependent upon age and circumstance	Vitamin A deficiency is rare in the United States. It is, however, a major issue in developing countries where the majority of research has been performed While Vitamin A is contained in various products marketed for immune support, it is not commonly sold as a single-ingredient product with such claims, and hence, this ingredient was given a lower rating for priorities to address through future research in the United States, within the scope of the systematic review research questions posed	4.0
MAD	1.38	1.10			0.66
LoA	U	A			U
**Vitamin C**					
Median	6.5	7.0	Studies show that taking vitamin C prophylactically may be effective in reducing severity or duration of illness that may occur, for otherwise healthy individuals (males) experiencing extreme physical or mental stress	*What's needed to say more* • RCT research involving competitive athletes, soldiers in training, long-distance travelers, medical students, under extreme physical and mental stress • Vitamin C given prophylactically in the range of 0.5–2 g/day; vitamin C provided prophylactically vs. provided at the first onset of a cold symptom (comparison); dose studies • Compared with placebo • Outcomes including incidence, severity, and duration of acute respiratory tract infection(s), immune function, and overall well-being • Duration up to 3 months *Barriers to research and resource needs* • Given the abundant source of vitamin C in diets, people may have varied baseline levels; challenge to control for both the baseline levels and habitual intake during trials • Need for RDA and DV to be re-evaluated for vitamin C recommendations • Individuals may have different needs; biotolerance differs across different people and circumstances or stressors • Pharmacological vs. physiological range for vitamin C dose/amount	7.0
MAD	1.13	0.50			0.81
LoA	U	A			U
**Vitamin D**					
Median	5.5	7.0	Few studies show benefit in taking vitamin D weekly or daily throughout the winter months among children and adults with various baseline levels Few studies in athletes or during military training show some benefit	*Barriers to research and resource needs* • Baseline serum levels not always known at study entry or categorization of adequate vs. inadequate, insufficient or deficient varies across studies with definitions not clear • Given the recent results of VITAL study, do vitamin D serum levels have to be rethought? • Unique populations such as military in environments with little sunlight, submariners, or space force should be investigated	6.5
MAD	1.75	1.28			1.25
LoA	U	U			U
**Vitamin E**					
Median	5.0	3.5	One study showed a greater risk of experiencing an infection, episodes, and duration of illness among healthy seniors. Other reviews suggest no benefit for the general healthy population	*Barriers to research and resource needs* • Need for clinically validated functional biomarkers • Different types/forms of vitamin E • Different results for different cohorts • Vitamin E more likely to be included in a multivitamin dietary supplement rather than as a single ingredient product for immune support	4.0
MAD	1.41	0.88			0.91
LoA	U	U			U
**Zinc**					
Median	8.0	8.0	Studies show that zinc supplementation may reduce the incidence, frequency, and durations of infections if taken prophylactically among children during winter season, U.S. cadets exposed to stressors and seniors, some of whom were zinc deficient	*What's needed to say more* • More RCT research on stressed individuals or exposed to winter season, to confirm effect; zinc-deficient, malnutrition-prone populations (e.g., elderly, children) • Standardized formulation for relevance to consumer options (tablets vs. lozenge vs. nasal sprays); doses in the range of replacement to correct a deficiency vs. a physiological dose • Compared with placebo • Outcomes including number of infections per group, severity, and duration; immune function • Duration up to 3 months; up to one year *Barriers to research and resource needs* • Optimal dose not understood • Various zinc formulations/delivery systems of lozenges, sprays, OTC drugs, homeopathic and dietary supplement tablets/capsules • Need for screening participants to identify those who are deficient or not • Blood zinc levels may not well represent those in targeted tissues/cells • Challenges of obtaining dietary intakes • Defined functional biomarkers for zinc status, beyond plasma levels • People are at risk for inadequate zinc status as society moves toward more plant-based foods	8.0
MAD	1.91	0.56			0.44
LoA	U	A			A

^a^ Priorities for research were rated on a 1–9-point scale with 1–3 representing low priority, 4–6 some priority, and 7–9 high priority for research.

^b^ After rerating the priority for research, panelists were asked to rate whether they believed it was appropriate to invest resources in clinical trial research specifically given the barriers discussed, at this time; 1–9-point scale with 1–3 representing absolutely inappropriate, 4–6 neutral, and 7–9 absolutely appropriate.

^c^ LoA defined as (A) Agreement, meaning ≤2 panelists outside the 3-point region containing the median (1–3, 4–6, 7–9), (D) Disagreement, meaning 3 or more panelists in each extreme (1–3 and 7–9), (U) Uncertainty meaning anything outside the above categories.

DV, daily value(s); RCT, randomized clinical trial.

## Discussion

The research expert panel process allowed for the formulation of recommendations to develop an innovative, robust research agenda based on what stakeholders reveal as the most pressing opportunities and/or barriers. The proposed next steps for the field will help facilitate meaningful research priorities and allow for the translation of dietary supplement research findings into useful information for consumers, health professionals, researchers, and policymakers. As we transition toward a society focused on health promotion and learning about resilience rather than focusing solely on disease prevention and treatment, these recommendations will assist with uncovering the requisite evidence needed to either support or not support the immune claims made on dietary supplement labels.

## Disclaimer

The opinions and assertions expressed herein are those of the author(s) and do not reflect the official policy or position of the Uniformed Services University or the Department of Defense. The contents of this publication are the sole responsibility of the author(s) and do not necessarily reflect the views, opinions, or policies of the Henry M. Jackson Foundation for the Advancement of Military Medicine. Mention of trade names, commercial products, or organizations does not imply endorsement by the Office of Dietary Supplements, National Institutes of Health, Department of Defense, nor the U.S. Government.
